# Hypoattenuated Leaflet Thickening After TAVR: Incidence, Predictors, and the Role of Platelet Reactivity: A Prospective Multicenter Observational Study

**DOI:** 10.3390/jcm15124469

**Published:** 2026-06-09

**Authors:** Pilar Jimenez-Quevedo, Carolina Espejo-Paeres, Francesco Spione, Breda Hennessey, Angela McInerney, Luis Marroquin, Esther Bernardo, Mª. Aránzazu Ortega-Pozzi, Gabriela Tirado-Conte, Fernando Macaya, Beatriz Cabeza, Irene Martín Lores, Pablo Salinas, Ivan Nuñez-Gil, Hernán Mejía-Rentería, Antonio Fernández-Ortiz, Jose Juan Gómez De Diego, Julián Perez-Villacastin, Javier Escaned, Ana Bustos, Manel Sabate, Alberto de Agustin Loeches, Nieves Gonzalo, Luis Nombela-Franco, Ander Regueiro, Eduardo Pozo Osinalde

**Affiliations:** 1 Cardiology Department, Clinico San Carlos University Hospital, Health Research Institute of Hospital Clínico San Carlos (IdISSC), 28040 Madrid, Spain; carolina.espejo.paeres@gmail.com (C.E.-P.); breda_82@hotmail.com (B.H.); angela_mcinerney@hotmail.com (A.M.); luismarroquin92@gmail.com (L.M.); bernardogarcia@yahoo.es (E.B.); aopozzi@hotmail.com (M.A.O.-P.); gabrielatirado@gmail.com (G.T.-C.); fernando.macaya.ten@gmail.com (F.M.); beatrizcabeza66@gmail.com (B.C.); irenemartinlores@gmail.com (I.M.L.); salinas.pablo@gmail.com (P.S.); ibnsky@yahoo.es (I.N.-G.); hmejiarenteria@gmail.com (H.M.-R.); antonio.fernandezortiz@salud.madrid.org (A.F.-O.); josejgd@gmail.com (J.J.G.D.D.); jvillacastin@secardiologia.es (J.P.-V.); escaned@secardiologia.es (J.E.); ana.bustos@salud.madrid.org (A.B.); albertutor@hotmail.com (A.d.A.L.); nieves_gonzalo@yahoo.es (N.G.); luisnombelafranco@gmail.com (L.N.-F.); eduardopozoosinalde@yahoo.es (E.P.O.); 2 Cardiology Department, University Hospital Clínic, Institut d’Investigacions Biomèdiques August Pi i Sunyer (IDIBAPS), 08036 Barcelona, Spain; francesco.spio@gmail.com (F.S.); masabate@clinic.cat (M.S.); anderregueiro@gmail.com (A.R.)

**Keywords:** transcatheter aortic valve replacement, subclinical leaflet thrombosis, hypoattenuated leaflet thickening, clopidogrel resistance

## Abstract

**Background/Objectives**: Hypoattenuated leaflet thrombosis (HALT) is a frequent finding after transcatheter aortic valve replacement (TAVR). Although high residual platelet reactivity (HPR) increases thrombotic risk after coronary stent implantation, its role in HALT remains unclear. This study aimed to determine the incidence and predictors of HALT in TAVR patients treated with dual antiplatelet therapy, focusing on the impact of HPR. **Methods**: This was a prospective, multicenter observational study. Between June 2018 and February 2022 patients with symptomatic severe aortic stenosis undergoing successful TAVR and treated with dual antiplatelet therapy for 3 months were included. Platelet reactivity was assessed 1–3 months post-TAVR using either VerifyNow (72%) or the Multiplate Analyzer (28%). HALT was evaluated using contrast-enhanced multidetector computed tomography. **Results**: A total of 169 patients were included (mean age 81.5 ± 5 years; 51% female). The incidence of HALT was 22%. Independent predictors of HALT were self-expanding valve (OR 3.05; 95% CI, 1.30–7.14; *p* = 0.010). Protective factors included larger prosthesis size (OR 0.78; 95% CI, 0.65–0.93; *p* = 0.007), statin-treated dyslipidemia (OR 0.37; 95% CI, 0.16–0.88; *p* = 0.024), and higher creatinine clearance (OR 0.98; 95% CI, 0.96–1.00; *p* = 0.035). HALT incidence was similar in patients with and without HPR (23.7% vs. 33.6%; *p* = 0.29). No differences in clinical outcomes were observed at 1 year. **Conclusions**: HALT occurred in nearly one-quarter of TAVR patients treated with dual antiplatelet therapy and was unrelated to platelet reactivity. Valve characteristics, renal function, and statin-treated dyslipidemia were associated with HALT, highlighting the multifactorial nature of its development.

## 1. Introduction

Hypoattenuated leaflet thickening (HALT) following transcatheter aortic valve replacement (TAVR) was first described in 2013 [1]. Following this publication, several registries reported the occurrence of reduced aortic valve leaflet motion and HALT after TAVR [2,3,4,5]. The prevalence of hypoattenuated leaflet thickening (HALT) has been reported to range from 12% to 40%, depending on the type of bioprosthetic valve and the timing of contrast-enhanced multidetector computed tomography imaging [2,3,4,5]. The clinical significance of this entity remains a subject of ongoing debate. Makkar et al. reported an increased incidence of non-procedural transient cerebrovascular events—but not stroke—in patients with HALT [2]. Similarly, a recent meta-analysis encompassing 11,098 patients across 25 studies found that the presence of HALT was associated with a 2.6-fold higher risk of stroke or transient ischaemic attack [5]. Conversely, it has been hypothesized that the presence of HALT may impact long-term valve durability, a consideration of particular importance in younger patients. In this context, histopathological analyses of explanted transcatheter aortic valves with leaflet thickening and structural valve degeneration have demonstrated thrombus formation associated with fibrotic changes. Notably, thrombus was observed in conjunction with calcification in specimens obtained four years post-implantation [6,7]. These findings suggest that thrombus formation may contribute to various stages of bioprosthetic valve deterioration, potentially serving as a nidus for early calcification and degeneration. Nevertheless, prospective studies are necessary to confirm these findings. Recent evidence has identified several factors associated with the development of HALT. In general, oral anticoagulation (OAC) appears to confer a protective effect, whereas conditions linked to a heightened prothrombotic state have been associated with an increased risk of HALT [5]. High residual platelet reactivity (HPR) despite dual antiplatelet therapy (DAPT) is a well-established predictor of ischaemic events and mortality following coronary stent implantation [8,9,10]. Although data in transcatheter aortic valve replacement (TAVR) populations remain inconclusive [11,12]. The objective of this study was to assess the incidence of HALT in a cohort of TAVR patients treated with DAPT, and to identify clinical and biological factors associated with its occurrence, including the presence of HPR and markers of systemic inflammatory response.

## 2. Materials and Methods

This is a multicentre, prospective, observational study carried out between June 2018 and February 2022. Patients with severe symptomatic aortic stenosis who had undergone successful TAVR implantation according to Valve Academic Research Consortium-3 criteria [13] and were treated with DAPT (aspirin and clopidogrel) were included. Patients with a recent stroke < 14 days prior to TAVR, a documented allergy to aspirin and/or clopidogrel, ineligibility for DAPT for 3 months, or an indication for OAC were excluded. In addition, patients with documented severe thrombocytopenia (<50,000 platelets U/L), documented moderate or severe hepatic impairment, severe chronic renal insufficiency with creatinine clearance <30 mL/min, or patients unable to undergo post-TAVR multislice computed tomography or to attend scheduled study follow-up visits were also excluded. The study was approved by the central ethics committee (17/250-E_BS). All patients provided specific written informed consent for this study prior to enrolment, in addition to informed consent for the procedure. All patients received aspirin (100 mg) and clopidogrel (300 mg loading dose) before TAVR, followed by aspirin 100 mg/day and clopidogrel 75 mg/day for 3 months. The loading dose of clopidogrel was not given if the patient was already receiving clopidogrel therapy.

### 2.1. Study Objectives

The primary objective of the study was to determine the factors associated with HALT in patients treated with DAPT for 3 months. Secondary objectives were: to assess the impact of HPR on the occurrence of clinical events, including death, myocardial infarction, cerebrovascular events, heart failure and bleeding, up to 1-year follow-up; to evaluate whether the systemic inflammatory response after TAVR, as measured by CD14+ and CD16- quantification, was associated with the occurrence of HALT; and to determine the incidence of clinical events during follow-up according to VARC-3. All patients underwent clinical follow-up at 1, 3, 6 months and 1 year. Patients who developed HALT were treated with oral anticoagulation at the discretion of the physician.

### 2.2. Platelet Reactivity Analyses

Platelet reactivity was measured using the Multiplate analyser (28% of the patients) (Dynabyte Informations system, Munich, Germany) or the VerifyNow P2Y12 assay (72% of the patients) (Accumetrics, San Diego, CA, USA) at 1–3 months (mean 64 + 25 days) after TAVR. The presence of HPR was defined according to the cut-off of each assay: ≥46 units for Multiplate or ≥208 platelet reactivity units for VerifyNow [14].

The VerifyNow P2Y12 assay is a whole-blood point-of-care method that mimics turbidometric aggregation and utilizes disposable cartridges containing 20 µM ADP and 22 nM PGE1. Aggregation testing using ADP as a sole agonist activates P2Y1 and P2Y12 purinergic signalling, while adding PGE1 increases the specificity of the test for P2Y12 signalling. The VerifyNow P2Y12 assay reports the results as P2Y12 reaction units (PRU).

Multiplate analyser: Blood samples were collected in hirudin tubes and analysed using the Multiplate analyser according to the standard protocol. The Multiplate analyser evaluates the change in impedance caused by platelet adhesion to the sensing units, which consist of silver-coated electrodes. The agonists used were adenosin diphosphate (6.4 µM). A volume of 20 μL of ADPtest reagent (Roche Diagnostics, S.L, Sant Cugat del Vallès, Barcelona, Spain) was added to 300 μL of hirudin whole blood. Aggregation curves were recorded for 6 min and platelet aggregation was determined as the area under the curve using arbitrary units of aggregation (AU·min).

### 2.3. Inflammatory Response

The inflammatory response was measured as previously described [15]. Briefly, systemic inflammatory response was measured by quantification of CD14+ and CD16- and was determined by flow cytometry in whole blood at baseline (before TAVR), 2 days, and 1 and 6 months after TAVR. Quantitative variables were expressed as the percentage of cells labelled with the monoclonal antibody.

### 2.4. Multislice Computed Tomography

Multislice computed tomography was performed 3–6 months (121 ± 90 days) after TAVR. The study used GE Optima CT 660 and Philips Brilliance. After intravenous administration of 80–100 mL of iodinated contrast (Omnipaque, GE Oslo, Oslo, Norway), images were acquired in synchrony with the ECG using a specific retrospective protocol programmed with submillimetric image thickness, which allows assessment of leaflet mobility and thickening without dose modulation. Scans were ideally performed at a heart rate of less than 70 bpm, with beta-blocker administration where possible. Scans were programmed at 120 kV, except in specific situations requiring higher voltage, such as dense stent TAVR, the presence of a permanent pacemaker, the presence of mechanical prostheses, or patients with an increased body mass index. Images were analysed offline using dedicated software (Extended Philips Brilliance Workspace). All scans were centrally reviewed by a single investigator (E.P.) who was blinded to all patient-related information. The leaflets were evaluated using two-dimensional multiplanar reconstructions and three-dimensional reconstructions of volume rendering. Parameters were analysed as follows: presence of HALT (quantitative assessment), maximum leaflet thickness, presence of leaflets with reduced motion (qualitative assessment of leaflet mobility). Leaflet motion was classified as normal, mildly reduced (<50% reduction in opening relative to the centre of the stent), moderately reduced (50–70% reduction in motion) and severely reduced (>70% reduction in motion) or immobile [16]. In addition, we evaluated the following: placement of the prosthesis, relationship with the orientation of the native commissures, TAVR depth below the native aortic annulus, implant asymmetry, stent measurements (major, minor, mean axis, perimeter and eccentricity index at the entry level of the native valve annulus and at the mid-level of the sinuses of Valsalva). Post-deployment THV dimensions (outer stent frame area and short- and long-axis diameter) were evaluated at 3 levels: inflow, midportion, and outflow. THV eccentricity was determined as 1−(minimum THV diameter/maximum THV diameter). The THV was deemed noncircular if eccentricity was >10% at all 3 levels. THV expansion was defined as THV area multislice computed tomography/THV area nominal. THV underexpansion was defined as an expansion ratio of ≤90% at all 3 levels [16].

### 2.5. Statistical Analyses

Quantitative variables are expressed as mean ± SD and qualitative variables as numbers and percentages. Discrete variables were compared using the chi-squared or Fisher exact test. A logistic regression model was constructed to identify independent predictors of HALT. Clinically and anatomically relevant parameters and variables with a *p*-value < 0.05 in the univariate analysis were entered into the model, which was built using stepwise backward regression with an elimination probability of 0.10. Variables included in the model were: dyslipidaemia, creatinine clearance, self-expanding valve and valve size. Based on the limited number of published reports in this area, the sample size was calculated by estimating a probability of valve thrombosis of 12% [2]; expecting an absolute increase in the HPR group of 18%, an alpha error of 0.05 and a beta error of 0.20, a minimum of 33 patients in each group was estimated. The frequency of HPR based on the previous literature was assumed to be 22% [8], so a sample size of 150 was calculated. A 10% increase for attrition was added, so a final size of 165 was achieved (37 HPR and 128 non-HPR). To assess how accurately PRU (VerifyNow) can diagnose HALT, we conducted a Receiver Operating Characteristic (ROC) analysis. The ROC analysis provided the full spectrum of potential cutoff values for the test (PRU) and its ability to differentiate between leaflet thrombosis and non-thrombosis. To gauge the predictive performance of the ROC analysis in this context, we computed the F1-score. The F1-score is derived from the test’s precision and recall: precision represents the proportion of true-positive results out of all predicted positives (including incorrect predictions), while recall indicates the proportion of true-positive results out of all actual positives. In biomedicine, precision is referred to as positive predictive value (PPV), and recall is termed sensitivity. The F1-score, which is the harmonic mean of precision and recall, provides a balanced metric that equally considers both. The formula for the F1-score is F1 = 2 × S × PPV/(S + PPV). An F1-score of 1.0 represents perfect precision and recall, while an F1-score of 0 indicates no predictive power.

## 3. Results

### 3.1. Baseline Characteristics

**Figure 1:**
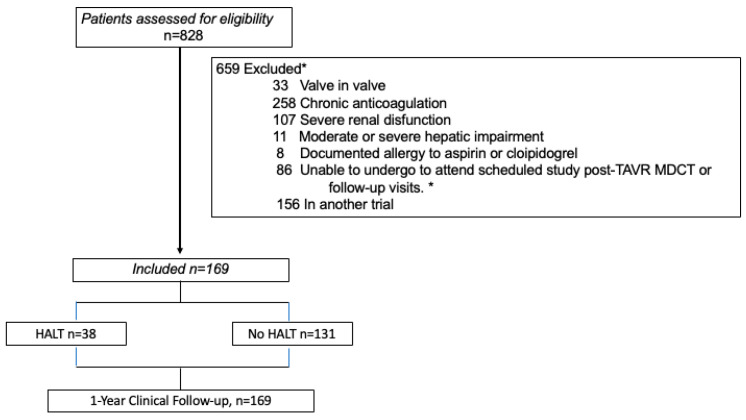
Flow chart of the study. * Multislice computed tomography or blood sample analysis could not be performed due to the COVID-19 pandemic.

From June 2018 to February 2022, a total of 169 patients were enrolled; the mean age was of 81.5 ± 5 years, 51% were female, and the mean STS score was 4.0 ± 5.8. The flow chart of the study is depicted in Figure 1. All procedures were performed via transfemoral access and 58.6% used a balloon-expandable valve. Predilatation was used in 36.1% and postdilatation in 11.2% of patients.

### 3.2. Subclinical Valve Thrombosis

**Figure 2:**
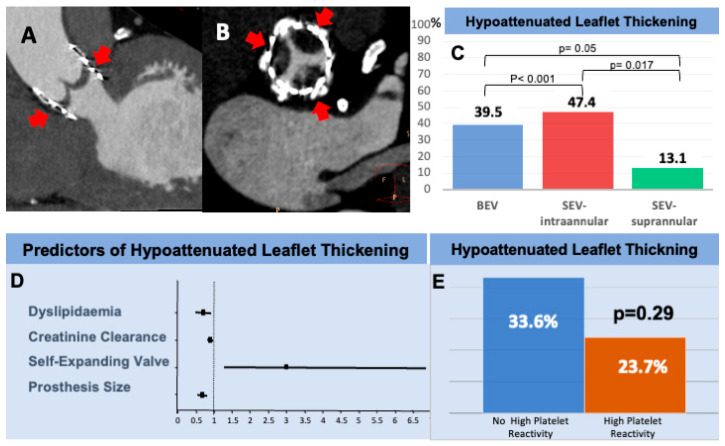
The occurrence of HALT according to valve type. (**A**,**B**): An example of a computed tomography image performed 3 months after transcatheter valve replacement in a patient with hypoattenuated leaflet thickening in the three cusps (red arrows). (**A**): Long-axis view; (**B**): short-axis view. (**C**): Incidence of hypoattenuated leaflet thickening by valve type implanted. BEV: Balloon-expandable valve; SEV: self-expanding valve. (**D**): Predictors of hypoattenuated leaflet thickening. (**E**): Incidence of subclinical leaflet thrombosis in patients with high residual platelet reactivity compared with patients without high residual platelet reactivity.

The overall rate of HALT was 22% (*n* = 38), observed at a mean of 121 ± 90 days after valve implantation. Patients with HALT had a lower body mass index, were less likely to have hyperlipidaemia treated with statins and had poorer renal function as assessed by creatinine clearance. In terms of procedural characteristics, HALT was more common in patients treated with a self-expanding valve, especially intra-annular and smaller valve sizes. The occurrence of HALT according to valve type is shown in the Figure 2. Baseline characteristics, echocardiographic parameters and procedural features are shown in Table 1, Table 2 and Table 3 respectively.

**Figure 3:**
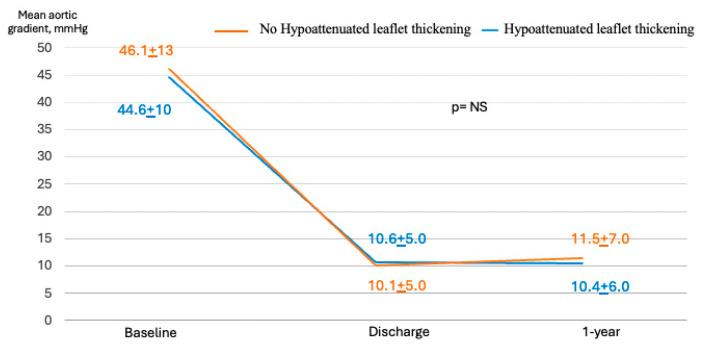
Temporal changes in mean aortic gradient measured by echocardiography from baseline, hospital discharge and 1-year follow-up.

Regarding CT parameters (median 112 (94–150) days), patients with HALT had less calcified aortic valves, smaller aortic annuli and smaller sinus of Valsalva dimensions (Table 4). The number of thrombosed leaflets was 1.65 ± 0.71 and the mean thickness was 5.4 ± 3.0 mm. Temporal changes in mean aortic gradients measured by echocardiography are shown in Figure 3.

Independent predictors of HALT were intra-annular self-expanding valve (odds ratio [OR] 4.20, 95%CI 1.71–10.29, *p* = 0.002); conversely, independent predictors of absence of HALT were larger prosthesis size (OR 0.81, 95%CI 0.67–0.98, *p* = 0.028) and the presence of dyslipidaemia treated with statins (OR 0.39, 95%CI 0.16–0.92, *p* = 0.032) and higher creatinine clearance (OR 0.98; 95% CI, 0.96–1.00; *p* = 0.035). In this context, increasing the prosthesis size by one unit reduces the risk of thrombosis by 22%. One-year clinical follow-up (mean 13.2 ± 9.0 months) was completed in all patients. At 1-year, patients with HALT did not exhibit any increase in major adverse cardiac, bleeding or cerebrovascular events (Table 5).

Of the 38 patients with HALT, 24 patients (63%) were treated with anticoagulation: 6 patients with vitamin K antagonist (acenocumarol), 2 patients with subcutaneous enoxaparin 1 mg/kg adjusted for renal function and 16 patients with non-vitamin K antagonist (7 patients with apixaban and 9 patients with edoxaban). All treated patients but one (*n* = 23) underwent CT at 1 year, showing a reduction in thrombus burden in 100% and complete resolution of HALT in 19 patients (82.6%). Of the remaining patients who were not anticoagulated (*n* = 14, 37%), one patient died of non-cardiac causes and the remaining patients had no clinical events at 1-year.

### 3.3. Platelet Reactivity and Inflammation

A total of 54 patients (32%) had HPR after DAPT following TAVR. These patients were more likely to be insulin-dependent diabetics, to have peripheral vascular disease, and to be frail according to the Fried classification. They also had a higher body mass index and lower haemoglobin levels (Table 6). Overall, the percentage of patients with HPR measured at 1 month was 33.8% (*n* = 54) with no differences seen between patients with and without HALT: 23.7% vs. 33.6%; *p* = 0.29. In-hospital and 1-year outcomes were similar in patients with and without HPR (Table 7). Independent predictors of HPR in patients treated with TAVR were: insulin-dependent diabetes, peripheral vascular disease, and body mass index and HB levels (Table 6).

We performed an exploratory analysis to determine the HPR cut-off value that best defines HALT in patients with TAVI. A PRU cut-off of 77 yielded the highest F1-score (0.4), making it the most balanced threshold for optimizing both sensitivity and positive predictive value in identifying patients with subclinical leaflet thrombosis. These results can be considered hypothesis-generating and would require further investigations in larger cohorts of patients.

**Figure 4:**
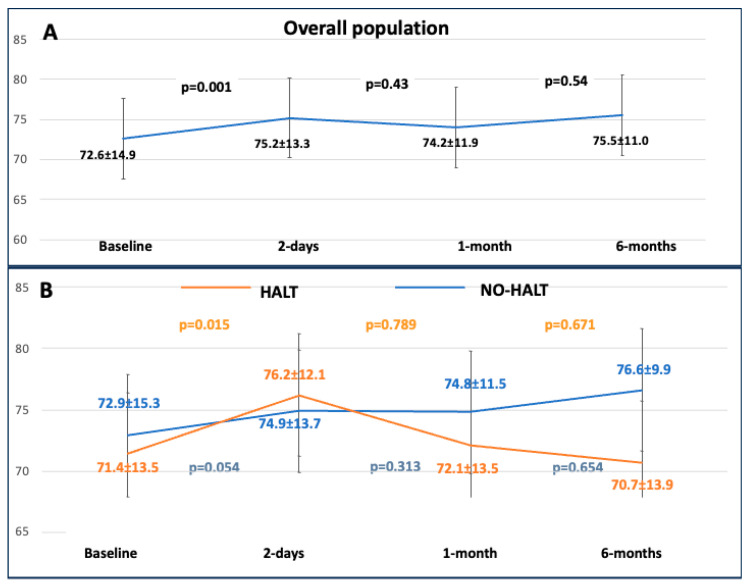
Temporal analyses of circulating inflammatory markers defined as CD14+ and CD16-. (**A**): Total population. (**B**): Temporal analyses of circulating inflammatory markers in patients with hypoattenuated leaflet thickening and patients without hypoattenuated leaflet thickening.

Circulating inflammatory markers showed a significant increase between baseline and 2 days after TAVR in the entire population (Figure 4A), although no differences were found at other time points during follow-up. Patients with HALT showed a numerically higher increase in the number of circulating CD14+ and CD16- between baseline and 2 days (from 72.9 ± 15.3 to 74.9 ± 13.7 in the non-HALT group and, from 71.4 ± 13.5 to 76.2 ± 12.1 in the HALT group), shown in Figure 4B.

## 4. Discussion

The main findings of the study were as follows: (1) among TAVR patients treated with DAPT, HALT was identified in approximately one-quarter of the population, while high residual platelet reactivity (HPR) was observed in one-third of patients; (2) no significant association was found between the presence of HALT and HPR; (3) independent predictors of HALT included implantation of an intra-annular self-expanding valve and smaller prosthesis size and poor renal function whereas the presence of dyslipidemia treated with statins was associated with a reduced risk; and (4) circulating inflammatory biomarkers exhibited distinct kinetic patterns between patients with and without HALT.

### 4.1. Systemic Inflammation and Platelet Reactivity

According to Virchow’s triad, the development of vascular thrombosis is a multifactorial process involving endothelial injury, abnormal blood flow, and alterations in haemostasis [7]. In this context, comorbidities such as obesity, hypertension, chronic obstructive pulmonary disease, smoking history, and renal dysfunction, conditions often associated with systemic inflammation, have been implicated in the pathogenesis of HALT [3,8].

Consistent with prior reports, this study found that markers of systemic inflammation, including advanced stages of chronic kidney disease, were associated with an increased risk of HALT. Interestingly, the presence of hyperlipidemia was inversely associated with HALT. Notably, all patients with hyperlipidemia were receiving statin therapy, which allows for the hypothesis that the anti-inflammatory properties of statins may confer a protective effect against HALT development. Supporting this notion, we observed a numerically greater rise in inflammatory biomarkers in patients with HALT, suggesting a potential link between systemic inflammation and leaflet thrombosis.

This study also demonstrated that high residual platelet reactivity (HPR) despite dual antiplatelet therapy (DAPT) was not associated with the incidence of HALT during follow-up. Two previous studies have investigated this relationship following TAVR and, consistent with our findings, reported no significant association between platelet reactivity and HALT occurrence [11,12]. However, several methodological differences distinguish those studies from ours. First, both included patients receiving chronic oral anticoagulation, which was a predefined exclusion criterion in our cohort and may partly account for the higher incidence of HALT observed in our population. Second, the timing of HPR assessment varied notably. In the study by Jiménez et al. platelet function was measured at 24 h, 72 h, and at discharge, with multislice computed tomography performed at a median of 114 days [12]. In contrast, Nührenberg et al. obtained blood samples at the beginning of the TAVR procedure and performed multislice computed tomography five days post-procedure [11]. These differences are important, as clopidogrel-induced platelet inhibition is known to vary over time, with clopidogrel resistance decreasing by up to 50% between days 1–5 and day 30. Similarly, the timing of multislice computed tomography imaging may influence the observed incidence of HALT, with studies reporting higher prevalence rates at one year compared to earlier time points [5].

Furthermore, recent clinical trials have provided robust evidence supporting single antiplatelet therapy (SAPT) as the preferred regimen following TAVR [17]. Contrasting with the pathophysiology of stent thrombosis in coronary interventions, where platelet activity is central, the finding that platelet reactivity did not significantly correlate with HALT indicates that subclinical leaflet thrombosis may be driven heavily by local stasis (Virchow’s triad) rather than purely systemic, platelet-mediated pathways [18].

### 4.2. CT-Based Evidence on HALT Across Clinical Trials and Surgical Risk Profiles

Regarding HALT data from randomized trials comparing TAVR versus surgery, dedicated CT-based studies in high-risk patients treated with self-expanding valves remain scarce. In this context, the PORTICO IDE CT analysis provides relevant information on subclinical leaflet thrombosis in high-risk patients undergoing TAVR. In this study, subclinical leaflet thrombosis was detected by four-dimensional CT in 22 of 55 patients (40%) approximately 1 month after TAVR. According to valve type, subclinical leaflet thrombosis was observed in 16 of 37 Portico valves (43%), 6 of 14 Sapien XT valves (43%), and in none of the 4 CoreValve prostheses. All cases showed corresponding hypoattenuating leaflet opacities on CT, consistent with subclinical leaflet thrombosis [19]. These data are in line with the findings of the present study, in which intra-annular self-expanding and balloon-expandable valves showed the highest rates of HALT, highlighting the concept that the occurrence of HALT may differ substantially according to valve design.

By contrast, most randomized CT substudy data comparing TAVR with SAVR come from low-risk populations. In the PARTNER 3 CT substudy, which compared balloon-expandable SAPIEN 3 TAVR with SAVR in low-risk patients, HALT was more frequent after TAVR than after SAVR at 30 days (13% vs. 5%; *p* = 0.03), whereas this difference was no longer significant at 1 year (28% vs. 20%; *p* = 0.19) [20]. Similarly, in the Evolut Low-Risk CT substudy, which compared supra-annular self-expanding TAVR with SAVR, HALT was observed in 17.3% of patients treated with TAVR and in 16.5% of patients treated with SAVR at 30 days. At 1 year, HALT increased to 30.9% after TAVR and 28.4% after SAVR, with no significant difference between groups [21].

Taken together, these CT substudies indicate that HALT is a dynamic phenomenon during the first year after bioprosthetic aortic valve implantation and may occur after both transcatheter and surgical procedures. Importantly, in both PARTNER 3 and Evolut Low-Risk, HALT was not associated with impaired valve haemodynamics at either 30 days or 1 year [20,21].

### 4.3. Mechanics, Valve Selection, and Sizing Optimization

The type of valve has been identified as a factor influencing the development of HALT, with distinct anatomical or haemodynamic factors contributing to risk in each case. The different cell designs and materials may play a role in blood stagnation between the prostheses and the sinuses of Valsalva [22].

The physical design of balloon-expandable and self-expanding valves creates fundamentally different geometric and haemodynamic conditions within the aortic root after implantation, with direct implications for neosinus flow dynamics, leaflet shear stress, and thrombogenic risk. The intra-annular position of balloon-expandable platforms and intranular self-expandable valves results in a larger neosinus volume and greater spatial separation between the prosthetic leaflets and the anatomic sinuses of Valsalva. Computational fluid dynamics studies have consistently demonstrated that this intra-annular configuration generates higher blood stagnation in the neosinus, leading to an increased thrombosis risk compared to supra-annular configurations [23]. In contrast, supra-anular self-expanding valves facilitate more effective blood washout and reduce the residence time that promotes thrombus formation [22,24].

In a meta-analysis by Bogyi et al. the presence of HALT was more common in intra-annular self-expanding valves, followed by intra-annular balloon-expandable valves, suggesting that this valve position increases the risk of HALT formation compared to supra-annular valves [5]. These results are consistent with the findings of the present study, in which the intra-annular self-expanding valve was associated with increased rates of HALT, with 47% of all HALT occurring in intra-annular self-expandable prostheses.

Beyond the prosthesis type, there are several haemodynamic factors which also play a critical role in the development of intravascular thrombosis, as regions of turbulent flow may facilitate platelet adhesion to the prosthetic valve surface and delay endothelialisation [25]. Previous studies have identified smaller valve area, elevated transvalvular gradients, severe prosthesis–patient mismatch, and smaller TAVR prosthesis size as risk factors associated with the development of HALT [26,27,28,29]. In agreement with these findings, our study demonstrated an inverse association between valve size and the presence of HALT. Smaller prostheses are more likely to generate higher residual transvalvular gradients and disturb physiological laminar flow, thereby promoting turbulence and thrombogenicity [26]. These findings underscore the importance of optimizing valve design to enhance haemodynamic performance, potentially reducing both the incidence of HALT and the risk of early structural valve deterioration.

Conversely, other reports have suggested that larger prosthesis size and wider sinuses of Valsalva may increase the risk of HALT. Notably, these studies primarily included balloon-expandable valves or identified both balloon-expandable design and larger prosthesis size as independent predictors of HALT [30,31,32]. Further studies are warranted to elucidate the specific anatomical features and prosthesis characteristics associated with HALT across different TAVR platforms.

Sizing optimization is also essential to reduce the risk of HALT after TAVR. Previous studies have shown that transcatheter heart valve deformation is associated with an increased risk of leaflet thrombosis. THV underexpansion may increase blood stasis on the surface of the valve leaflets and promote platelet activation, with experimental data showing a linear relationship between the degree of underexpansion and blood stasis [33]. This concept has also been supported by in vivo CT studies demonstrating an association between prosthesis deformation and the occurrence of HALT after TAVR [34].

Conversely, excessive oversizing or overexpansion may also promote HALT. In patients undergoing TAVR with SAPIEN 3 valves, oversizing greater than 20% was independently associated with an increased risk of HALT [18]. Mechanistically, excessive radial force may distort the prosthetic frame, reduce neosinus volume, and induce asymmetric leaflet expansion, all of which can promote local flow stasis [18,34].

Taken together, these findings suggest that both insufficient and excessive prosthesis expansion may create unfavourable haemodynamic conditions that predispose to HALT. Therefore, improved sizing strategies avoiding both underexpansion and excessive oversizing may represent an important procedural approach to reduce leaflet thrombosis after TAVR. In this regard, the development of additional intermediate prosthesis sizes may also be helpful, as it could allow more precise matching between annular anatomy and valve dimensions, reducing the need for excessive oversizing or accepting suboptimal expansion in borderline annular measurements.

All this data enhances the importance of CT preprocedural planning to predict the risk of HALT. In this regard, the neosinus has emerged as a key anatomical substrate for HALT. Mechanistic and computational studies have supported the concept that impaired blood washout and increased residence time within the neosinus may promote thrombus formation on transcatheter valve leaflets [35]. More recently, artificial intelligence and computational fluid dynamics applied to preprocedural CT have shown promising performance for HALT prediction, supporting the evolving role of CT from an anatomical planning tool to a platform for individualized thrombotic risk stratification, device selection, procedural optimization, and potentially post-TAVR antithrombotic management [36].

### 4.4. HALT, Valve Haemodynamics, and Stroke

Although the clinical significance of HALT remains debated, its potential association with haemodynamic valve deterioration and cerebrovascular events has raised concern. Long-term observational studies, such as those by Rashid et al. and Hein et al., have demonstrated a significant association between early CT-detected HALT and subsequent haemodynamic valve deterioration [18,37]. However, more recent long-term data have not confirmed this relationship. In a cohort of patients undergoing TAVI with SAPIEN XT or SAPIEN 3 and early post-procedural MDCT, Iwata et al. found that HALT detected within 30 days was not associated with long-term clinical outcomes, haemodynamic valve performance, or structural valve deterioration beyond 6 years [38]. Similarly, the relationship between HALT and stroke remains controversial. A meta-analysis of 25 studies including 11,098 patients reported that subclinical leaflet thrombosis after TAVR was associated with a 2.6-fold higher risk of stroke or TIA [5]. However, this finding has not been consistently confirmed in individual studies or randomized CT substudies, such as the PARTNER 3 and Evolut Low-Risk CT substudies in which stroke event rates were low and HALT was not associated with a significant increase in cerebrovascular events [20,21]. Thus, although HALT may identify a subgroup of patients at increased thromboembolic risk, whether it is a causal determinant of stroke remains uncertain [27,39,40].

### 4.5. Limitations

This study is subject to the inherent limitations of an observational design. At the time the study protocol was developed, clinical guidelines recommended dual antiplatelet therapy (DAPT) following TAVR. However, during the recruitment period, these recommendations evolved to favour single antiplatelet therapy (SAPT). Although previous studies have reported no significant difference in HALT incidence between SAPT and DAPT regimens [4], the findings of this study may not be generalizable to patients managed with SAPT or oral anticoagulation (OAC) post-TAVR. Formal patient–prosthesis mismatch was not systematically assessed in this study. Therefore, the potential contribution of PPM to HALT development could not be specifically evaluated and should be addressed in future studies. While the study was initially designed to include a consecutive series of patients meeting predefined inclusion and exclusion criteria, the enrollment process was ultimately non-consecutive due to the disruptions caused by the COVID-19 pandemic. Furthermore, although HALT was not associated with adverse clinical events in our cohort, the study was underpowered to detect differences in hard clinical endpoints such as stroke or mortality. These negative findings regarding the relationship between HRP and HALT are hypothesis-generating and larger, prospective studies are needed to validate these observations and assess their clinical implications.

## 5. Conclusions

In this study, the incidence of HALT among patients undergoing successful TAVR and treated with DAPT was relatively high yet not associated with adverse clinical outcomes at 1-year follow-up. Several clinical, anatomical, and procedural factors were associated with the development of HALT, whereas high residual platelet reactivity (HPR) was not. The absence of a relationship between platelet activity and HALT raises an important question regarding the utility of antiplatelet therapy in this setting. Nevertheless, definitive conclusions cannot be drawn, and further studies are needed to evaluate the safety of minimizing or withholding antiplatelet agents in TAVR patients. Additionally, differences in the kinetic profiles of circulating inflammatory biomarkers between patients with and without HALT suggest a potential role of systemic inflammation in its pathogenesis. However, due to the limited sample size, these negative findings regarding the relationship between HRP and HALT are hypothesis-generating and future mechanical exploration using computational fluid dynamics paired with longitudinal clinical registries is required to definitively decouple systemic clotting factors from focal mechanical stasis.

## Figures and Tables

**Table 1:** Baseline characteristics.

Baseline Characteristics				
	Overall (*n* = 169)	No HALT (*n* = 131)	HALT (*n* = 38)	*p* Value
Age (year)	82 ± 5	82 ± 5	83 ± 5	0.205
Female *n* (%)	95 (51%)	61 (41%)	24 (63%)	0.072
Weight (kg), *n* (%)	72.3 ± 13.3	73.9 ± 13.1	66.9 ± 12.6	0.004
Height (cm), *n* (%)	160.8 ± 9.3	161.6 ± 9.3	158.1 ± 9.5	0.046
Body mass index (kg/m^2^)	28.0 ± 4.9	28.4 ± 5.0	26.7 ± 4.4	0.078
Hypertension, *n* (%)	128 (68.8%)	103 (78.6%)	25 (65.8%)	0.163
Diabetes mellitus *n* (%)	65 (34.9%)	55 (42.0%)	10 (26.3%)	0.099
Dyslipemia, *n* (%)	107 (57.5%)	89 (67.9%)	18 (47.4%)	0.031
Current smoker, *n* (%)	41 (24.3%)	36 (27.5%)	5 (13.2%)	0.081
History of stroke or TIA, *n* (%)	20 (11.8%)	15 (11.5%)	5 (13.2%)	0.732
Peripheral vascular disease, *n* (%)	12 (7.1%)	9 (6.9%)	3 (7.9%)	0.796
Renal insufficiency, *n* (%)	30 (17.8%)	23 (17.6%)	7 (18.4%)	0.849
Gastrointestinal disorder, *n* (%)	17 (10.1%)	13 (9.9%)	4 (10.5%)	0.874
Pulmonary disease, *n* (%)	29 (17.2%)	24 (18.3%)	5 (13.2%)	0.494
Previous myocardial infarction, *n* (%)	17 (10.1%)	13 (9.9%)	4 (10.5%)	0.874
Previous CABG, *n* (%)	7 (4.1%)	6 (4.6%)	1 (2.6%)	0.614
Previous PCI, *n* (%)	35 (20.7%)	28 (21.4%)	7 (18.4%)	0.745
Previous mitral-valve surgery, *n* (%)	0 (0%)	0 (0%)	0 (0%)	-
Angina *n* (%)	39 (23.1%)	26 (19.8%)	13 (34.2%)	0.064
NYHA functional class III-IV *n* (%)	55 (32.5%)	46 (35.1%)	9 (23.7%)	0.217
Fragility *n* (%)	86 (50.9%)	69 (52.7%)	17 (44.7%)	0.666
STS score	4.0 ± 5.8	4.0 ± 6.5	3.7 ± 1.7	0.754
EuroSCORE II	4.0 ± 4.5	4.1 ± 4.8	3.5 ± 2.9	0.531
EuroSCORE log	12.9 ± 10.1	12.5 ± 9.9	14.3 ± 10.9	0.345
Laboratory parameters				
Creatinine levels (mg/dL)	1.0 ± 0.3	1.0 ± 0.3	0.9 ± 0.3	0.831
Creatinine clearance by Cockcroft–Gault formula (ml/min/m)	63.8 ± 22.9	65.9 ± 23.7	56.3 ± 18.5	0.025
Haemoglobin levels (g/dL)	12.9 ± 1.6	12.8 ± 1.7	13.2 ± 1.4	0.157
Platelets levels (×10^9^/L)	209.7 ± 56.8	212.4 ± 57.8	199.6 ± 52.7	0.284
NTproBNP (pg/mL)	2246.9 ± 3675.9	2360.6 ± 3744.2	1881.6 ± 3477.6	0.515
Baseline therapy				
Aspirin, *n* (%)	60 (35.1)	50 (37.6)	10 (26.3)	0.200
Clopidogrel, *n* (%)	6 (3.6%)	5 (3.8%)	1 (2.6%)	0.747
Statins, *n* (%)	107 (57.5%)	89 (67.9%)	18 (47.4%)	0.031
Renin–angiotensin system inhibitor, *n* (%)	96 (56.2%)	75 (57.2%)	21 (55.3%)	0.99

HALT: Hypoattenuated leaflet thickening; TIA: transient ischaemic attack; CABG: coronary artery bypass graft; PCI: percutaneous coronary intervention; NYHA: New York Heart Association; STS score: Society of Thoracic Surgeons score; NTproBNP: N-terminal brain natriuretic peptide.

**Table 2:** Echocardiographic parameters.

Echocardiographics Parameters				
	Overall (*n* = 169)	No HALT (*n* = 131)	HALT (*n* = 38)	*p* Value
Mean aortic gradient (mmHg)	47.0 ± 14.2	47.2 ± 14.2	46.6 ± 14.2	0.826
Maximum peak aortic gradient (mmHg)	76.5 ± 21.9	77.0 ± 22.6	74.8 ± 19.3	0.589
Left ventricular ejection fraction, (%)	58.3 ± 11.6	57.6 ± 11.5	60.6 ± 11.7	0.167
Moderate–severe pulmonary hypertension, *n* (%)	26 (15.4%)	21 (16.0%)	5 (13.2%)	0.715
Bicuspid valve, *n* (%)	4 (2.4%)	3 (2.3%)	1 (2.6%)	0.908
Mitral regurgitation	117 (69.6%)	24 (64.9%)	93 (71.0%)	0.474

HALT: Hypoattenuated leaflet thickening.

**Table 3:** Procedural features.

Procedural Features				
	Overall (*n* = 169)	No HALT (*n* = 131)	HALT (*n* = 38)	*p* Value
Transfemoral access	169 (100%)	131 (100%)	38 (100%)	>0.99
Balloon-expandable valve Self-expanding intra-annular valve Self-expanding supra-annular valve	99 (58.6%) 36 (21.3%) 34 (20.1%)	84 (64.1%) 18 (13.7%) 29 (22.4%)	15 (39.5%) 18 (47.4%) 5 (13.2%)	0.013 <0.001 0.224
Valve size	25.9 ± 2.6	26.1 ± 2.6	25.1 ± 2.6	0.05
Aortic balloon pre-dilatation, *n* (%)	61 (36.1%)	46 (35.1%)	15 (39.5%)	0.928
Aortic balloon post-dilatation *n* (%)	19 (11.2%)	13 (7.7%)	6 (15.8%)	0.305
Post-dilatation balloon size (mm),	22.9 ± 1.8	23.2 ± 1.6	22.2 ± 2.2	0.319

HALT: Hypoattenuated leaflet thickening.

**Table 4:** Computerized tomography parameters at baseline and at follow-up.

Baseline Parameters	Overall (*n* = 169)	No HALT (*n* = 131)	HALT (*n* = 38)	*p* Value
Agatston score	2888.0 ± 1457.1	3046.9 ± 1494.3	2352.2 ± 1193.6	0.013
Maximum aortic annulus diameter (mm)	26.1 ± 3.3	26.5 ± 3.4	24.9 ± 2.6	0.013
Minimal aortic annulus diameter (mm)	21.6 ± 2.7	21.9 ± 2.8	20.6 ± 2.4	0.011
Mean aortic annulus diameter (mm)	24.1 ± 2.4	24.4 ± 2.4	23.4 ± 2.4	0.023
Aortic annulus area (mm^2^)	450.1 ± 95.7	462.1 ± 96.1	407.3 ± 82.0	0.001
Aortic annulus perimeter (mm)	74.8 ± 9.0	75.7 ± 9.2	71.6 ± 7.1	0.016
Left coronary ostium height (mm)	12.6 ± 2.8	12.8 ± 2.9	11.9 ± 2.4	0.103
Right coronary ostium height mm	14.1 ± 3.2	14.3 ± 3.3	13.3 ± 2.4	0.102
**No coronary Valsalva sinus diameter (mm)**	29.4 ± 3.8	29.7 ± 3.8	28.37 ± 3.68	0.057
Right Valsalva sinus diameter (mm)	29.6 ± 3.9	29.9 ± 3.9	28.54 ± 3.71	0.055
Left Valsalva sinus diameter (mm)	30.7 ± 3.7	30.9 ± 3.6	29.75 ± 3.61	0.072
Mean Valsalva sinus diameter (mm)	29.9 ± 3.6	30.2 ± 3.7	28.9 ± 3.5	0.050
Parameters at 3–6 Months Follow-up				
Leaflet movility (%)	91.9 ± 16.6	99.35 ± 4.0	65.5 ± 17.1	<0.001
Commisural alignment (º)	29.6 ± 14.8	29.7 ± 14.5	29.0 ± 16.0	0.800
Circumferential expansion *n* (%)	116 (68.6%)	88 (67.2%)	28 (73.7%)	0.435
Prothesis depth (mm) (media ± SD)	6.9 ± 2.3	6.9 ± 2.3	7.1 ± 2.6	0.610
Aortic annulus/Valsalva sinus diameter	0.98 ± 0.05	0.98 ± 0.05	0.98 ± 0.01	0.565
Prothesis–right Valsalva sinus length (mm)	5.1 ± 1.9	5.0 ± 1.9	5.3 ± 1.6	0.058
Prothesis–left Valsalva sinus length (mm)	5.8 ± 2.1	5.8 ± 2.0	5.7 ± 2.1	0.668
Prothesis–non-coronary Valsalva sinus length (mm)	5.0 ± 1.9	5.1 ± 1.8	4.8 ± 2.0	0.246
Mean prothesis–Valsalva sinus length	5.3 + 1.6	5.3 + 1.7	5.3 + 1.5	0.893
Left ventricular outflow tract excentricity (%)	9.9 ± 6.9	9.5 ± 6.9	11.1 ± 7.0	0.193
Aortic annulus excentricity (%)	8.2 ± 6.1	8.3 ± 6.3	8.0 ± 5.4	0.780
Valsalva sinus excentricity (%)	7.4 ± 4.9	7.5 ± 4.9	6.9 ± 4.8	0.504
Prosthesis expansion at LVOT (%)	88.1 ± 18.2	88.8 ± 16.1	85.6 ± 24.4	0.354
Prosthesis expansion at aortic annulus (%)	86.5 ± 14.9	86.9 ± 14.3	85.2 ± 16.9	0.537
Prosthesis expansion at Valsalva sinus	90.6 ± 16.7	90.7 ± 15.9	90.2 ± 19.6	0.863

HALT: Hypoattenuated leaflet thickening.

**Table 5:** In-hospital and at 1 year follow-up clinical events.

In-Hospital Outcomes				
	**Overall** **(*n* = 169)**	**No HALT** **(*n* = 131)**	**HALT (*n* = 38)**	***p***
Annulus rupture *n* (%)	0 (0%)	0 (0%)	0 (0%)	-
Vascular complication *n* (%)	12 (7.1%)	10 (7.6%)	2 (5.3%)	0.616
Major vascular complication *n* (%)	2 (1.2%)	2 (1.5%)	0 (0%)	0.444
Major bleeding *n* (%)	2 (1.2%)	1 (0.8%)	1 (2.6%)	0.340
Pacemaker inplantation, *n* (%)	24 (14.2%)	18 (13.7%)	6 (15.8%)	0.750
Coronary occlusion, *n* (%)	1 (0.6%)	1 (0.8%)	0 (0%)	1.000
Stroke, *n* (%)	3 (1.8%)	2 (1.5%)	1 (2.6%)	0.650
Renal insufficiency, *n* (%)	1 (0.6%)	1 (0.8%)	0 (0%)	1.000
Myocardial infarction, *n* (%)	1 (0.6%)	1 (0.8%)	0 (0%)	1.000
All-cause death, *n* (%)	0 (0%)	0 (0%)	0 (0%)	-
1-year Outcomes				
	**Overall (*n* = 169)**	**No HALT** **(*n* = 131)**	**HALT** **(*n* = 38)**	***p* value**
All-cause death *n* (%)	10 (5.9%)	8 (6.1%)	2 (5.3%)	0.846
Myocardial infarction *n* (%)	1 (0.6%)	1 (0.8%)	0 (0%)	0.585
Stroke *n* (%)	6 (3.6%)	4 (3.1%)	2 (5.3%)	0.533
Bleeding *n* (%)	8 (4.7%)	7 (5.3%)	1 (2.6%)	0.475
Mean aortic transprosthetic gradient (mmHg)	10.5 ± 4.7	10.9 ± 4.7	9.1 ± 4.4	0.111

HALT: Hypoattenuated leaflet thickening.

**Table 6:** Univariate analyses according to the presence or not of high residual reactivity.

	No High Platelet Reactivity (*n* = 106)	High Platelet Reactivity (*n* = 54)	Odd Ratio(OR) (Univariate)	*p* Value	Adjusted OR	*p* Value
Age, y	81.4 ± 5.7	82.1 ± 4.4	0.99 (0.93–1.05)	0.655		
Body Mass Index (Kg/m^2^)	27.4 ± 4.7	29.5 ± 5.3	1.08 (1.01–1.16)	0.017	1.11 (1.03–1.19)	0.007
Women, *n* (%)	50 (47.2)	32 (59.2)	1.63 (0.84–3.16)	0.147		
Hypertension, *n* (%)	78 (73.5)	47 (87.0)	2.41 (0.98–5.95)	0.115		
Hyperlipidemia, (*n* %)	66 (62.3)	34 (62.9)	1.03 (0.52–2.03)	0.931		
Diabetes Mellitus, *n* (%)	36 (34.0)	30 (55.5)	2.43 (1.24–4.75)	0.009		
Haemoglobin Levels, gr/dL	13.1 + 1.7	12.3 + 1.4	0.71 (0.57–0.89)	0.003	0.68 (0.54–0.87)	0.002

**Table 7:** In-hospital and 1-year clinical events in patients with high platelet reactivity compared to patients without high platelet reactivity.

In-Hospital Clinical Events						
	No HPR (*n* = 106)	HPR (*n* = 53)	Odds Ratio (IC95%)	*p* Value	Adjusted OR	*p* Value
All cause death,	0 (0%)	0 (0%)	-		-	
Myocardial infarction	0 (0%)	0 (0%)	-		-	
Stroke,	2 (2.7%)	0 (0%)	-		-	
Major bleeding	1 (0.9%)	5 (9.3%)	10.7 (1.22–94.1)	0.032	1.66 (0.05–57.3)	0.781
Major vascular complication	5 (4.7%)	1 (1.9%)	0.38 (0.04–3.35)	0.384	0.26 (0.02–3.44)	0.256
Renal injury	0 (0%)	0 (0%)	-			
Pacemaker	12 (16.4%)	14 (15.4%)	0.92 (0.40–2.14)	0.854	0.95 (0.40–2.26)	0.946
Clinical events at 1-year follow-up						
	No-HPR	HPR	Hazard Ratio (IC95%)	*p* value	Adjusted HR	*p* value
Death	7 (6.6%)	3 (5.7%)	1.78 (0.45–7.10)	0.415	0.85 (0.17–4.26)	0.846
Cardiac death	1 (0.9%)	1 (1.9%)	6.79 (0.32–146.1)	0.221	30.7 (0.00–9999)	0.803
Myocardial infarction	0 (0%)	1 (1.9%)	-		-	
Stroke	4 (3.8%)	2 (3.8%)	1.10 (0.20–5.99)	0.916	0.61 (0.08–4.37)	0.620
Major bleeding	6 (5.7%)	1 (1.9%)	0.53 (0.06–4.56)	0.563	0.19 (0.01–2.75)	0.221
MACE	10 (9.4%)	4 (7.5%)	1.55 (0.57–4.20)	0.394	0.60 (0.16–2.22)	0.449

MACE: Major adverse clinical events; HPR: high platelet reactivity.

## Data Availability

The data presented in this study are available on request from the corresponding author. The data are not publicly available due to privacy concerns.

## Abbreviations

The following abbreviations are used in this manuscript:

TAVR	Transcatheter aortic valve replacement
HPR	High residual platelet reactivity
DAPT	Dual antiplatelet therapy
HALT	Hypoattenuated leaflet thickening
